# Pre-Sleep Cognitive Arousal Is Negatively Associated with Sleep Misperception in Healthy Sleepers during Habitual Environmental Noise Exposure: An Actigraphy Study

**DOI:** 10.3390/clockssleep4010010

**Published:** 2022-02-24

**Authors:** Rachel L. Sharman, Michael L. Perlis, Célyne H. Bastien, Nicola L. Barclay, Jason G. Ellis, Greg J. Elder

**Affiliations:** 1 Nuffield Department of Clinical Neurosciences, Sleep and Circadian Neuroscience Institute, University of Oxford, Oxford OX3 9DU, UK; rachel.sharman@ndcn.ox.ac.uk; 2 Behavioral Sleep Medicine Program, Perelman School of Medicine, University of Pennsylvania, 3535 Market Street, Philadelphia, PA 19104, USA; mperlis@upenn.edu; 3 École de Psychologie, Université Laval, Québec, QC G1V 0A6, Canada; celyne.bastien@psy.ulaval.ca; 4 Centre de Recherche CERVO, Québec, QC G1E 1T2, Canada; 5 Sleep Universal Ltd., Oxford OX1 2JD, UK; nicola.l.barclay@gmail.com; 6 Northumbria Sleep Research, Department of Psychology, Faculty of Health and Life Sciences, Northumbria University, Newcastle upon Tyne NE1 8ST, UK; jason.ellis@northumbria.ac.uk

**Keywords:** actigraphy, environmental noise, sleep misperception, pre-sleep arousal

## Abstract

Specific noises (e.g., traffic or wind turbines) can disrupt sleep and potentially cause a mismatch between subjective sleep and objective sleep (i.e., “sleep misperception”). Some individuals are likely to be more vulnerable than others to noise-related sleep disturbances, potentially as a result of increased pre-sleep cognitive arousal. The aim of the present study was to examine the relationships between pre-sleep cognitive arousal and sleep misperception. Sixteen healthy sleepers participated in this naturalistic, observational study. Three nights of sleep were measured using actigraphy, and each 15-s epoch was classified as sleep or wake. Bedside noise was recorded, and each 15-s segment was classified as containing noise or no noise and matched to actigraphy. Participants completed measures of habitual pre-sleep cognitive and somatic arousal and noise sensitivity. Pre-sleep cognitive and somatic arousal levels were negatively associated with subjective–objective total sleep time discrepancy (*p* < 0.01). There was an association between sleep/wake and noise presence/absence in the first and last 90 min of sleep (*p* < 0.001). These results indicate that higher levels of habitual pre-sleep arousal are associated with a greater degree of sleep misperception, and even in healthy sleepers, objective sleep is vulnerable to habitual bedside noise.

## 1. Introduction

Sufficient good quality sleep is crucial for maintaining positive physical and psychological health [1]. Sleep loss, or sleep disruption, is associated with a wide range of negative health outcomes, including weight gain, diabetes, cardiovascular disease, and depressive disorders [1,2].

It is well established that noise can disrupt subjective and objective sleep [3], as indicated by laboratory studies and naturalistic studies which have been conducted in habitual sleep environments [3,4]. There is evidence of a dose–response relationship between noise and both subjective sleep disturbances and objective cortical arousal during sleep [4]. For example, one laboratory study demonstrated that relative to quiet nights, noisy nights resulted in increased fatigue and lower subjective sleep quality [5]. This study also showed that noisy nights resulted in objective changes to sleep including reduced sleep efficiency, reduced total sleep time (TST), increased wake after sleep onset (WASO) and an increased latency to slow-wave sleep (SWS) [5]. Additionally, the negative impact of noise upon subjective sleep is increased if the noise stimulus is a combination of different sounds [6]. Taken together, noise can lead to negative effects upon subjective and objective sleep, although less is known regarding the impact of representative daily household noise upon sleep [7].

Enhanced information processing, particularly at sleep onset and during sleep, can result in a discrepancy between perceived subjective sleep, and the objective sleep actually attained, whereby individuals underestimate their TST (i.e., resulting in “sleep misperception”) [8,9]. Individuals with insomnia disorder often report worse subjective sleep continuity (sleep latency (SL), WASO and TST) than is observed with objective measures [10,11,12]. While this occurs to variable degrees, in its extreme (important discordances between subjective and objective measures of SL and/or WASO and/or TST), this is referred to as sleep misperception or Paradoxical Insomnia [13]. This sleep misperception may be related to the presence of high-frequency electroencephalography (EEG) activity during sleep, representing cortical arousal [14]. Even healthy sleepers may experience increased sensory processing, and sleep disturbances, in a noisy environment. This is evidenced by the fact that environmental noise can elicit cortical arousals in healthy sleepers [15].

There are very likely to be intra-individual factors which influence the level of disruption caused to sleep by external environmental noise, whereby some individuals are more vulnerable to the effects of nocturnal bedside noise upon sleep than others. One specific factor is elevated levels of pre-sleep cognitive arousal, which refers to worrying about sleep, being unable to stop thoughts whilst attempting to sleep, or having an overly-active or racing mind [16]. It is well documented that increased levels of pre-sleep cognitive arousal have been associated with subjective difficulties in falling asleep in a range of adults without insomnia disorder [17,18,19,20,21]. A large body of evidence strongly indicates that people with insomnia disorder experience more pre-sleep cognitive arousal than healthy sleepers [22].

Pre-sleep cognitive arousal can affect the degree of sleep misperception: a prospective actigraphy study of individuals with insomnia disorder indicated that higher levels of pre-sleep cognitive arousal predicted the degree of objective total sleep time underestimation [23]. This is also the case in non-insomnia populations: individuals with higher levels of pre-sleep cognitive arousal typically estimate lower total sleep times, and longer sleep onset latencies, relative to objective measurements [24]. The discrepancy between subjective and objective sleep can be quantified by calculating the percentage of objective sleep estimated (OSE%). An OSE% percentage value of <100% indicates that the subjective estimate of TST is lower than the actual obtained TST (which is indicative of poor perceived sleep quality), and a percentage value of >100% indicates that the subjective TST is greater than obtained TST (which is indicative of good perceived sleep quality) [13].

However, it is not known if pre-sleep cognitive arousal is related to sleep misperception, or objective awakenings, in the context of habitual bedside noise. Subjective noise sensitivity may also influence noise-induced sleep disturbances: one sleep laboratory study, where air, rail and traffic noises were played to participants, showed that subjective noise sensitivity was associated with subjective sleep disturbances [25]. Similarly, it is not known if subjective noise sensitivity can influence the subjective–objective sleep discrepancy.

Whilst there is a strong association between environmental noise and sleep disruption, few studies have measured the impact of typical neighbourhood noises such as voices, pets, and footsteps in the room, which are heard in the bedroom, upon the sleep of healthy sleepers [26,27]. Although these noises are typically lower in volume than the 45-decibel limit which is considered to be detrimental [4], if salient (personally meaningful), this may cause shallow sleep, arousals, or short or long awakenings; indeed; sound pressure levels which are as low as 33 dBA can still exert physiological effects during sleep [4,28]. Therefore, they are potentially likely to create sleep misperception, even in healthy sleepers.

Furthermore, there are specific points of sleep where healthy sleepers may be vulnerable to the effects of habitual bedside noise. Objective sleep is typically comprised of four to five ultradian sleep cycles of approximately 90 min in duration, which alternate between NREM and REM sleep [29]. Most NREM sleep is obtained in the first half of the night, and REM sleep is more present in the last third of the night [30]. Cortical arousals are typically structured and are not randomly-distributed [31]. Even in healthy sleepers, up to 40% of individuals report experiencing wakefulness prior to forced awakenings from NREM sleep, and up to 20% of individuals report experiencing wakefulness prior to forced awakenings from REM sleep [32]. Additionally, these individuals reported having an awareness of the external environment, despite being objectively asleep [32]. Even in typical sleepers, the first 2 h of NREM sleep and the last hour of sleep appear to be more susceptible to feelings of awakening prior to a forced awakening from sleep [33].

The overall aim of the present study was to examine the relationships between pre-sleep cognitive arousal and sleep misperception. Specific aims of the study were to assess (1) associations between habitual pre-sleep arousal and sleep misperception, (2) if bedside noise was associated with objective nocturnal wake, and finally, (3) if the first 90 min of sleep (when there is a predominance of NREM-SWS) is potentially more susceptible to noise disruption than the last 90 min (when there is a predominance of REM) in the night, in a habitual home environment in healthy sleepers. Whilst actigraphy does not have the specificity of polysomnography, particular advantages of this method include the low burden placed upon participants and good cost-effectiveness and is consequently an ideal method of evaluating sleep in a naturalistic environment [34]. Therefore, in the context of the present study, measuring sleep using actigraphy provides extremely high levels of ecological validity.

The present study had the following specific hypotheses:

1. Habitual pre-sleep cognitive arousal will be negatively associated with the subjective–objective total sleep time discrepancy (i.e., a higher habitual pre-sleep arousal will be associated with a lower OSE% value, indicating a greater degree of sleep misperception).
2. Habitual pre-sleep cognitive arousal will be positively associated with the total number of objectively-measured epochs of waking associated with noise (but not waking with no noise, or sleep with noise, or sleep with no noise).
3. Subjective noise sensitivity will be negatively associated with the subjective–objective total sleep time discrepancy (i.e., a higher noise sensitivity will be associated with a lower OSE% value, indicating a greater degree of sleep misperception).
4. The presence of bedside noise will be associated with objective waking in both the first and last 90 min of sleep.
5. Nocturnal noise will result in more objective waking than sleep during the first 90 min of sleep, relative to the last 90 min.

## 2. Results

Demographic and relevant questionnaire results are shown in Table 1. A total of four participants had estimated objective sleep (OSE%; see Section 4.10) values of <100% which indicated that these participants had a high degree of sleep misperception, as their subjective estimate of TST was lower than the TST that was actually achieved. Twelve participants had OSE% values of >100%, indicating that they had a low degree of sleep misperception, as their subjective estimate of TST was higher than the TST that was actually achieved (Table 1). Subjective and objective summary sleep data are displayed in Table 2.

There was a significant negative relationship between PSAS pre-sleep cognitive arousal and OSE% scores (r = −0.66, *p* < 0.01), and also between PSAS pre-sleep somatic arousal and OSE% scores (r = −0.61, *p* < 0.025); specifically, this indicated that greater pre-sleep arousal was associated with a greater degree of sleep misperception, to the extent that participants estimated ‘better’ sleep as compared to that objectively measured. There was no significant relationship between OSE% values, representing sleep-state misperception, and WNSS scores, representing subjective noise sensitivity (r = −0.34; *p* > 0.05).

Pre-sleep cognitive arousal (PSAS) was not significantly associated with the total number of objective epochs of wake associated with noise (r = −0.01, *p* > 0.0125). Pre-sleep cognitive arousal was also not significantly related to the total number of objective epochs of wake associated with wake with no noise, sleep associated with noise, or sleep associated with no noise (all *p*-values > 0.0125). Pre-sleep somatic arousal was not associated with the total number of objective epochs of wake associated with noise, wake with no noise, sleep associated with noise, or sleep associated with no noise (all *p*-values > 0.0125).

There was a significant association between sleep/wake and noise presence/absence in both the first 90 min of sleep (χ^2^ (1,5768) = 49.84, *p* < 0.001, ϕ = 0.09; Table 3) and the last 90 min of sleep (χ^2^ (1,5774) = 64.22, *p* < 0.001, ϕ = 0.11; Table 4). Odds ratios indicated that wake was 1.78 times more likely than sleep in noise conditions in the first 90 min of sleep, and 2.18 times more likely than sleep in noise conditions in the last 90 min of sleep.

There was a significant interaction between time point and noise status (Table 5) upon the percentage of awakenings (F(1.27, 38.04) = 4.51, *p* < 0.05; η^2^_p_ = 0.13), which indicated that awakenings in the first 90 min and last 90 min of sleep were differentially affected by noise. However, following corrections for multiple comparisons, post hoc comparisons of the percentage of awakenings observed at each time point were not significant (all *p*-values > 0.0125). There was a significant main effect of noise status (F(1.27, 38.04) = 70.35, *p* < 0.001; η^2^_p_ = 0.70). The main effect of time point was not significant (*p* > 0.05).

## 3. Discussion

The overall aim of the present observational study was to examine the relationships between pre-sleep cognitive arousal and sleep misperception, and pre-sleep cognitive arousal and objective awakenings in the presence of nocturnal, typical, environmental bedside noise. A secondary aim of the present study was to test the impact of routine environmental bedside noise upon subjective and objective sleep, and if the first 90 min of sleep is potentially more susceptible to noise disruption than the last 90 min in the night.

As expected, habitual pre-sleep cognitive arousal levels were negatively associated with the subjective–objective sleep discrepancy, which is a marker of sleep misperception. Specifically, higher levels of pre-sleep arousal were associated with a greater sleep discrepancy (i.e., where the subjective TST estimate was lower than the actual obtained TST). However, habitual pre-sleep cognitive arousal was not positively associated with the total number of objectively-measured epochs of wake associated with noise, and the degree of subjective noise sensitivity was not negatively associated with the subjective–objective TST discrepancy. As was hypothesised, environmental noise was associated with objective nocturnal wake, both in the first 90 min of sleep and in the final 90 min of sleep. However, unexpectedly, the first 90 min of sleep did not appear to be more susceptible to noise disruption than the final 90 min of sleep.

These results indicate that firstly, habitual pre-sleep cognitive arousal, but not subjective noise sensitivity, is associated with the degree of sleep misperception in healthy sleepers. These results are in line with previous work demonstrating that individuals with higher levels of pre-sleep cognitive arousal typically estimate lower total sleep times relative to objective measurements [24]. As the majority of participants over-estimated TST relative to objective sleep, this indicates that in terms of subjective sleep, overall, most participants can habituate to the presence of noise in their routine bedroom environment. This may reflect habituation because although subjectively, healthy sleepers can habituate to noise over time in experimental situations, objectively, their sleep remains disturbed [35]. Pre-sleep somatic arousal levels were also negatively associated with the degree of sleep misperception in healthy sleepers. Pre-sleep somatic arousal refers to physical symptoms including elevated heart rate, body tension or difficulty breathing [16]. This finding was not expected because although somatic arousal may have a role in the development of insomnia disorder [8,9], it is a difficult concept to measure, and self-reported measures of somatic arousal may instead reflect underlying anxiety [16,36]. Speculatively, this suggests that pre-sleep somatic arousal may also contribute to sleep misperception in the context of habitual beside noise, and this should be explored further.

Secondly, these results indicate that even in healthy sleepers, objective sleep is vulnerable to the effect of environmental habitual noise. Previous studies have examined the specific effects of disruptive sources of environmental noise, such as road traffic, air traffic, or wind turbines, and generally find negative effects upon sleep [3,4,5]. However, to the best of our knowledge, this is one of the first studies to measure the impact of everyday, habitual, nocturnal environmental noise upon actigraphically-measured objective sleep in healthy sleepers. This would indicate that even healthy sleepers have some degree of vulnerability to external environmental noise, even in their habitual nocturnal sleeping environment. This is supported by the apparent differences between subjective and objective sleep continuity in the present study, where participants reported uniformly better subjective sleep than objective sleep. Specifically, participants reported reduced SL, NWAK, WASO and better TST compared to what was objectively measured: this indicates that healthy sleepers perceive that they had better sleep than they objectively achieved. An alternative interpretation of these results is that as nocturnal body movements can be interpreted by actigraphy as an awakening [34], this might indicate that the more noise is present in the environment, the more body movements occur, and this may represent sleep fragmentation rather than outright wake. This may also explain the apparent discrepancy between subjective and objective NWAK and WASO in the present study. Alternatively, these results may be due to the lower overall awakening thresholds that are typically observed in REM sleep compared to NREM sleep [37], and future work should specifically assess if this is the case. Speculatively, there are two potential practical implications of the present study. Firstly, the present study demonstrates that even habitual environmental noise can disrupt objective sleep of healthy sleepers and that this effect is not limited to a particular time point. This suggests that maintaining a quiet bedroom environment, free from habitual environmental noise, is likely to maintain good sleep health. Secondly, interventions to reduce pre-sleep cognitive and somatic arousal are likely to lessen the disruptive impact of habitual environmental noise.

This study could be extended in three ways. Firstly, future studies could explore whether individuals with high habitual pre-sleep cognitive arousal levels display a greater degree of sleep misperception relative to individuals with low pre-sleep cognitive arousal in the presence of bedside noise. This could potentially be done using PSAS cognitive cut-off values which have been used to identify significant difficulties in initiating sleep and subjective insomnia symptoms [36]; similarly, this categorisation approach has been used to identify individuals at risk of stress-related vulnerability to sleep disturbances and assess their objective sleep and physiological arousal [38]. Secondly, it was beyond the scope of the present study to assess how pre-sleep cognitive arousal might specifically contribute to sleep misperception on a night-by-night basis. Thirdly, it was beyond the scope of the present study to categorise the emotional valence of specific sounds on each night: it is possible that personally-relevant or threatening sounds may have been more disruptive to sleep [28]; however, we have demonstrated that irrespective of the valence or noise source, bedside noise is still disruptive to objective sleep. Finally, it is possible that pre-sleep cognitive arousal, and habituation, may be situational: in experimentally-controlled noisy environments, whilst objectively, the sleep of healthy sleepers can be disturbed, subjective sleep demonstrates a degree of habituation [35,39].

Specific strengths of the study include the well-characterised nature of the participants, in that they were confirmed to be healthy sleepers free from relevant sleep or psychiatric disorders; additionally, the habitual environment allowed the potential impact of routine nocturnal noises upon objective sleep to be elucidated. Potential limitations of the present study include the correlational design used to explore the relationship between pre-sleep cognitive arousal, noise sensitivity and sleep misperception, and between pre-sleep cognitive arousal and objective periods of wake. However, this design was necessary to assess this relationship in a habitual home environment; future studies may wish to categorise participants into high and low-vulnerability groups as suggested, or alternatively, employ experimental designs to deliberately increase pre-sleep cognitive arousal, or manipulate noises. Similarly, objective sound pressure levels were not measured, and future studies may wish to incorporate this into their design. Finally, we were unable to specifically account for the type of participant residence in the present study. For instance, it is possible that a bedroom facing a road may experience more ambient noise than a bedroom facing a garden/yard area; similarly, we were unable to account for typical noise in the present study, and therefore, the noises experienced may have been additive. These factors were, however, beyond the scope of the present study and should be investigated further.

Overall, the results of the study indicate that in the presence of nocturnal, typical, environmental bedside noise in healthy sleepers, (1) higher levels of habitual pre-sleep arousal are associated with a greater degree of sleep misperception and (2) objective sleep is vulnerable to the effect of environmental habitual bedside noise.

## 4. Materials and Methods

### 4.1. Participants

A total of 16 healthy sleeper participants (M_age_ = 27.50 years, SD_age_ = 4.27 years) were recruited from a staff and student population and from a laboratory recruitment database containing members of the general public. Due to the exploratory nature of the present study, an a priori power analysis was not conducted.

Participants were eligible if they were (1) aged between 18 and 60 years of age; (2) lived in their own or rented accommodation; (3) had a stable sleep-wake pattern (assessed using a subjective sleep diary prior to study entry and defined as going to bed and awakening at consistent times during this period). Participants were not eligible if they: (1) lived in shared accommodation with the potential for excessive noise (e.g., university halls of residence); (2) reported current sleep problems, shift work, subjective anxiety or depression (assessed using the Hospital Anxiety and Depression Scale [40]), whereby scores ≥ 11 points were considered clinically-problematic) relevant physical illness, or were taking medication with the potential to affect sleep; (3) had a current hearing disorder; (4) reported transmeridian travel in the three months prior to the study.

Participants (and their bed partners, if appropriate) provided written informed consent, and the study was approved by Northumbria University Faculty of Health and Life Sciences ethics committee.

### 4.2. Materials and Measures

To measure the usual intensity of cognitive and somatic arousal immediately prior to sleep, participants completed the Pre-Sleep Arousal Scale (PSAS; [41]). The PSAS provides a subjective trait-like measure of habitual pre-sleep cognitive (e.g., being mentally alert) and somatic arousal (e.g., heart racing before sleep). Cognitive and somatic arousal subscale scores range from 8 to 40, where higher scores represent greater levels of pre-sleep arousal. As a measure of subjective noise sensitivity, participants completed the Weinstein Noise Sensitivity Scale (WNSS; [42]), where higher scores (potentially ranging from 0 to 126) represent greater subjective noise sensitivity.

### 4.3. Sleep Measurements

*Subjective sleep:* Consensus Sleep Diaries (CSD-M; [43]) were used to obtain measures of subjective sleep continuity: total sleep time (TST); time in bed (TIB), which refers to the period of time between the participant going to bed, and leaving the bed, on the subsequent morning; sleep efficiency (SE%), which was calculated as (TST/TIB × 100); sleep onset latency (SOL), as a measure of the length of time it took participants to fall asleep; the number of awakenings (NWAK), referring to the frequency of nocturnal awakenings during sleep; and wake after sleep onset (WASO), which refers to the duration of nocturnal time awake following the initiation of sleep.

*Objective sleep:* actigraphy was used to obtain objective sleep information, where accelerometers were continuously worn on the nondominant wrist (Actiwatch AW4, Cambridge Neurotechnologies, Cambridge, UK). Participants were instructed to press a marker button to indicate nightly time in bed and when the watch was removed for bathing/showering. Actigraphy data were analysed using accompanying manufacturer software (Actiwatch Activity and Sleep v7.4.3, Cambridge Neurotechnologies, Cambridge, UK) in order to algorithmically convert activity counts into sleep and wake and to derive standard measures of objective sleep continuity (TST, TIB, SE%, SOL, NWAK and WASO).

During the baseline period (Day −7 to 0), data were recorded in 60-s epochs, where epochs containing 40 accelerometer activity counts or more were automatically classified as wake, and fewer than 40 were automatically classified as sleep. During the observational period (Day 1 to 3), in order to provide greater levels of specificity, data were recorded in 15-s epochs, and due to the shorter recording window, epochs containing more than 10 counts were automatically classified as wake, and fewer than 10 counts as sleep.

### 4.4. Nocturnal Environmental Audio Recording

Nocturnal environmental noise from each participant’s usual bedroom environment during sleep was recorded using a high-quality digital audio recorder, with a built-in high-sensitivity three-directional microphone (Olympus LS-3, Olympus, Southend-on-Sea, UK). Participants were instructed to place the audio recorder as close to their head as possible without the recording device being placed on the bed or the floor. For example, participants were instructed that they could place the audio recorder device on a bedside table, or on an object of a similar height. Participants were asked to start the recording device immediately prior to attempting sleep and end this immediately upon awakening. Audio was recorded as 320 kbps MP3 files.

### 4.5. Procedure

**Figure 1:**
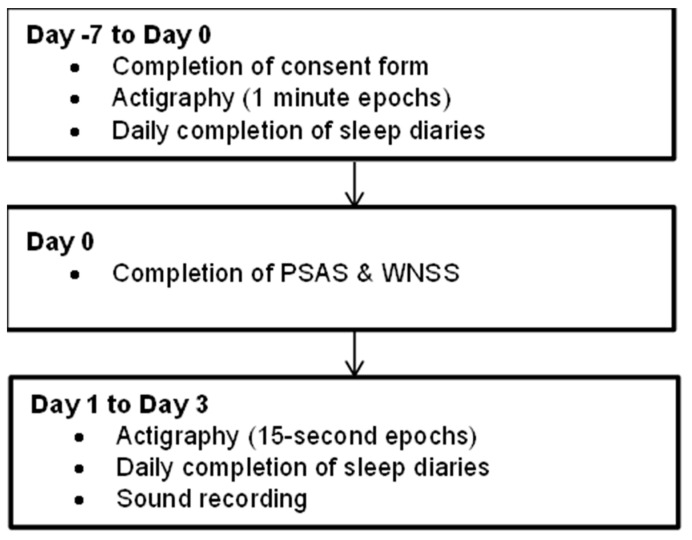
Study schematic.

This study was a ten-day ambulatory study conducted within participants’ routine, usual home environment and consisted of a baseline period (Day −7 to 0) and an observational phase (Day 0 to Day 3). The study procedure is summarised in Figure 1.

### 4.6. Baseline Period

Upon responding to the study invitation, participants were invited to attend the sleep laboratory. Participants then provided informed consent and were confirmed as a healthy sleeper by examining their sleep, psychiatric and physical illness history. Daily sleep diaries and simultaneous actigraphy were used, as described, to verify that participants were healthy sleepers over the next 7 days.

### 4.7. Observational Phase

On Day 0, participants returned to the sleep laboratory to complete the PSAS, WNSS and FIRST. Participants also collected the digital audio recorder and were shown how to use this. Participants were also asked to abstain from drinking alcohol during the observational phase of the study. From Day 1 to 3, participants were instructed to complete amended sleep diaries on each morning as described and to wear actigraphy accelerometers. On Night 1 to Night 3, participants were instructed to start the recording device at the point of lights out and to end the recording upon awakening. Participants were also instructed to press a marker button on the accelerometer at the same time. On Day 4, participants returned to the sleep laboratory for a debrief and received a payment of 30 GBP for their time.

### 4.8. Data Analysis

Baseline period data was used to verify that study participants were healthy sleepers and were not analysed further. Complete observational data were obtained from 16 participants. If a participant failed to press the marker button to indicate the beginning and end of the sleep recording period, sleep and wake times from the sleep diary were used as a substitute and were verified by inspecting digital audio file timestamps. Of the 96 recording period marker time points, *n* = 8 (8%) were substituted from sleep diaries.

### 4.9. Environmental Noise and Actigraphy Sleep Classification

In order to derive an objective number of nocturnal noise events, digital audio files were downloaded from the audio recorder and converted to WAV format using Audacity software (v.2.0.3, Audacity Team; https://www.audacityteam.org/, accessed 26 January 2022). This software was used to visually inspect the waveform of the audio recording. From this waveform, a short (5 s) period of relative silence at the beginning of the recording (i.e., a quiet period without any audible external noises) was used as a baseline level of environmental noise. This baseline noise level was subtracted from the full recording, which subsequently generated distinct noise peaks representing separate environmental noise events.

As the focus of the study was upon the first 90 min after sleep onset, and the last 90 min of sleep, Audacity was used to place markers at 15-s intervals of the audio recording, which corresponded with the 15-s actigraphy epochs, during these time points. This generated 360 (180 from the first 90 min, and 180 from the last 90 min) separate 15-s audio epochs, which were classified as either having environmental noise present or absent.

Actigraphy data from the same 360 15-s epochs were either classified as being “sleep” or “wake”. Overall, this meant that each 15-s epoch, from the first and last 90 min of sleep, could be classified as having wake with noise (WN), wake with silence (WS), sleep with noise (SN) and sleep with silence (SS). Total epoch counts were added together and averaged across the three observational nights for each participant.

### 4.10. Subjective-Objective Sleep Discrepancy

The discrepancy between subjective sleep and objective sleep was obtained by measuring the difference between sleep diary and actigraphy-measured TST. This was done by calculating the percentage of objective sleep estimated (OSE%) = (TST_sleepdiary_/TST_actigraphy_) × 100; a percentage value of <100% indicates that the subjective estimate of TST is lower than the actual obtained TST; a value of 100% indicates perfect concordance between subjective and objective TST, and a percentage value of >100% indicates that the subjective TST is greater than obtained TST [13].

### 4.11. Statistical Analyses

To examine the association between habitual pre-sleep cognitive and pre-sleep somatic arousal with sleep misperception, Pearson correlations were conducted between PSAS cognitive and somatic scores and OSE% values, with *p*-values adjusted for multiple comparisons (corrected *p*-value = 0.025).

Associations between habitual pre-sleep cognitive and pre-sleep somatic arousal with sleep misperception were assessed using Pearson correlations (PSAS cognitive and somatic scores, and OSE% values), with *p*-values adjusted for multiple comparisons (corrected *p*-value = 0.025).

A series of Pearson correlations were conducted between PSAS cognitive and PSAS somatic scores and the total number of epochs WN, WS, SN, SS, with *p*-values adjusted for multiple comparisons (corrected *p*-value = 0.0125). These were done to measure the relationship between habitual pre-sleep cognitive arousal and the total number of epochs of wake associated with noise, wake with no noise, sleep with noise, or sleep with no noise. The relationship between subjective noise sensitivity and sleep misperception was assessed using a Pearson correlation between WNSS and OSE% values.

To assess the association between sleep/wake and noise presence/absence in both periods, two chi-square tests were conducted (in the first 90 and last 90 min of sleep). In order to assess if nocturnal noise during the first 90 min of sleep would result in more objective wake than sleep, a mixed 2 (first 90 min vs. last 90 min of sleep) × 4 (noise status: WN, WS, SN, SS) analysis of variance (ANOVA) was conducted upon the averaged percentages of each epoch classification.

Finally, to explore the potential associations between habitual subjective arousal and stress levels, with sleep misperception, a series of Pearson correlations were conducted between PSS, PSRS, FIRST scores, and OSE% values, (corrected *p*-value = 0.017).

## Figures and Tables

**Table 1:** Participant demographics (*n* = 16).

	Mean	SD
Age	27.50	4.27
Gender (male/female; *n*/%)	8 (50%)/8 (50%)	
Shared property with other people (*n*/%)	14 (87.5%)	
Had bedpartner (*n*/%)	3 (19%)	
Had children in household (*n*/%)	2 (13%)	
Habitual accommodation type (*n*/%)	Apartment: 8 (50%) House: 8 (50%)	
Bedroom had windows (*n*/%)	16 (100%)	
Location of bedroom window (*n*/%)	Facing road: 9 (56%) Facing garden or yard: 7 (44%)	
OSE%	103.01	10.95
OSE% < 100% (*n*/%)	4 (25%)	
OSE% > 100% (*n*/%)	12 (75%)	
PSAS (cognitive)	18.69	5.30
PSAS (somatic)	9.81	2.20
WNSS	75.44	18.70

OSE%: percentage of objective sleep estimated; PSAS: Presleep Arousal Scale; WNSS: Weinstein Noise Sensitivity Scale.

**Table 2:** Summary of subjective and objective sleep from observational study phase (n = 16).

	Subjective Sleep		Objective Sleep	
	Mean	SD	Mean	SD
TIB (mins)	478.17	59.09	456.98	59.33
TST (mins)	417.81	53.05	407.50	47.01
SOL (mins)	11.73	10.37	19.17	15.06
NWAK	0.85	0.76	37.94	13.47
WASO (mins)	7.45	16.78	49.06	21.91
SE(%)	87.71	8.74	85.67	5.52

NWAK: number of awakenings; SE: sleep efficiency; TIB: time in bed; TST: total sleep time; WASO: wake after sleep onset.

**Table 3:** Number of 15-s actigraphy epochs classified as sleep or wake in the presence of noise or silence (first 90 min).

	Noise	Silence
Sleep	2748	2241
Wake	534	245

**Table 4:** Number of 15-s actigraphy epochs classified as sleep or wake in the presence of noise or silence (last 90 min).

	Noise	Silence
Sleep	3509	1398
Wake	733	134

**Table 5:** Percentage of each actigraphy epoch classification.

	WN		WS		SN		SS	
	Mean	SE	Mean	SD	Mean	SD	Mean	SD
First 90 min	9.25	6.79	4.25	4.17	47.61	18.98	38.78	17.68
Last 90 min	12.69	6.46	2.29	1.88	60.72	17.22	24.20	18.52

WN: wake with noise; WS: wake with silence; SN: sleep with noise; SS: sleep with silence.

## Data Availability

The data presented in this study are available upon reasonable request from the corresponding author. The data are not publicly available due to privacy reasons.
